# Intensity of Nematode Infection in Children Aged 3 to 5 Years Living in Mukuru Kwa Njenga Slum Settlement, Nairobi, Kenya

**DOI:** 10.1155/2020/4124808

**Published:** 2020-04-14

**Authors:** Lynda Allan, Fiona N. Mbai, Dorcas S. Yole, Moses Owino

**Affiliations:** ^1^Department of Biomedical Sciences and Technology, The Technical University of Kenya, P.O. Box 52428-00200, Nairobi, Kenya; ^2^Department of Applied Sciences and Technology, The Technical University of Kenya, P.O. Box 52428-00200, Nairobi, Kenya; ^3^Embakasi Medical Health Centre and Mukuru Health Centre, P.O. Box 30108-00100, Nairobi, Kenya

## Abstract

**Background:**

The burden of nematode infections is high mostly in children below 5 years old, with clinical manifestations ranging from mild to painful symptoms due to severe infections that end up suppressing the immune system of the infected children. The occurrence of these infections is highest in areas of extreme poverty. This study evaluated the intensity of nematode infections and assessed the status of deworming in children aged 3 to 5 years living in Mukuru slum settlement, Nairobi County, Kenya. *Methodology*. A total of 172 children aged between 3 and 5 years were sampled across the 5 major villages of Mukuru Slum settlement: Kwa Njenga, Vietnum, Wapewape, Kwa Reuben, and Motomoto. Community health workers administered questionnaires on the deworming history of children. Stool samples were collected, macroscopically examined, and microscopically analysed using Kato-Katz technique to assess the intensity of infection. The intensities of nematode infections were expressed as eggs per gram (epg) of faeces.

**Results:**

The point prevalence of nematode infection among the 98 children in the 1^st^ sampling was 25.5% with a mean infection intensity of 5424 epg, whereas among the 74 children sampled in 2^nd^ sampling, 47.3% had nematode infection with a mean infection intensity of 12384 epg. The average nematode infection for the 172 participants was 34.9% with a mean intensity of 17808 epg. The highest number of children infected with nematodes was in the village of Wapewape where 34 participants were examined and 36.3% were infected with a mean intensity of 3216 epg. Kwa Reuben and Vietnum villages had the same prevalence values of 32.4% where 34 participants in each village had a mean intensity of 3624 epg and 4512 epg, respectively. In both samplings, more than 80% of children had been dewormed more than 6 months prior to the study. *Ascaris lumbricoides* was the only species of intestinal nematodes identified to be present in the stool samples of children in this study, whereas *Trichuris trichiura* and hookworm infections were found to be absent. The intensity of infection was not dependent on age or gender.

## 1. Introduction

The rates of intestinal nematode infections are high in children. These parasites are largely infectious and their spread is linked with poverty. Therefore, their occurrence is highest in areas of poor hygiene [1]. There are several species of nematode worms; however, Hookworms, *Ascaris lumbricoides*, and *Trichuris trichiura* are among the most prevalent nematodes, estimated to infect almost one-sixth of the global population [2]. One significant feature of nematodes, given their expected global prevalence, is the link between the occurrence and strength of infection. While prevalence indicates the population affected, morbidity is dependent on infection intensity.

The symptoms indicating the presence of an intestinal nematode are commonly associated with the severity of infection [3]. A slight nematode infection is often asymptomatic, while a mild to heavy infection can be linked with painful and severe symptoms leading to suppression of the immune system. However, indirect damage to the immune system can occur in the absence of any noticeable infection by interfering with immune response processes to other infectious diseases.

The principal mechanisms by which intestinal nematodes damage human hosts are diverse taking into account feeding on host tissues, including blood, leading to a loss of iron and protein (especially with hookworm), resulting in maldigestion or malabsorption of nutrients [3]. This can result in stimulation of inflammatory responses that could affect appetite and food intake or change the metabolism and storage of key nutrients such as iron. This pathway causes typical responses to infection, such as fever and increased metabolic rate which end in immune responses to infection.

Infection with multiple gastrointestinal nematodes in children is widespread [4]. Pathologies associated with soil-transmitted helminthic infections may lead to acute illnesses, cognitive impairment, and sometimes long-term disability or early death [5]. Independently, it has been well documented that persistent infection with a particular nematode can impair physical and mental growth [6] and also affect the nutritional status and general development of children [7]. A study [8] in Mukuru found that 40.6% of 160 participants had stunted growth and the prevalence of wasting in Mukuru was at 13.3% and 30.5% for underweight children.

Parasites such as intestinal worm infections can contribute to undernutrition. Environments of poor hygiene and sanitation raise exposure of children to most parasites including nematode infestation [9]. This needs new approaches for the improvement and application of community control methods. Public-health participation is important for lasting control in a community [1]. The collective involvement comprises, but is not restricted to, delivery of clean water, communal health training, diet cleanliness practices, and upkeep of operational sanitation structures. Conversely, the application and sustainability of such involvement are demanded.

## 2. Materials and Methods

### 2.1. Ethical Approval

The Ethical Review Committee (ERC) analysed and approved this study on the basis of compliance with the committee's satisfaction with its scientific validity, justification, and relevance of purpose. Written and signed consent was obtained from the parents or guardians of all the children involved in the study. In addition to this, the Nairobi County Health Operational Research working group authorized the research to be conducted in the county. A permit to conduct this research was issued by the National Commission for Science Technology and Innovation.

### 2.2. Study Area

This study was carried out in Mukuru Kwa Njenga slum settlement of Nairobi County. Conditions in Mukuru are typical of slum settlements in Nairobi. It has a large poor population with no access to minimum services, living largely in structures made out of temporary and recycled building materials—or made out of timber, mud walling, and roofing made up of substandard materials such as sacks, carton paper, and polythene. There is no proper sanitation and waste management.

### 2.3. Sampling Method

The sample size was determined using baseline data obtained from the medical records of surveys and previous studies done in the study area within the past six months by the Division of Infectious Diseases in Children under the Ministry of Health prior to the study. The information from these previous related scientific studies was used to calculate the sample size using the formula by [11].

Community-based health workers assisted in obtaining informed and written consent from the parents or guardians of the children, mapping the study site, and administering questionnaires on status of deworming and symptoms of nematode infection in children.

Sample collection was done in five major villages known as “vijiji” within Mukuru Kwa Njenga. These sections included Kwa Njenga, Vietnum, Wapewape, Kwa Reuben, and Motomoto. At each village an equal number of male and female children were randomly selected across ages 3, 4, and 5 years. This was a cross-sectional study to assess the intensity of intestinal nematode among the sampled children.

### 2.4. Inclusion and Exclusion Criteria

Children aged 3 to 5 years living in Mukuru Kwa Njenga whose parents/guardians had given consent were included in this study. Children excluded from this study were those who were less than 3 years old and more than 5 years, those living outside of Mukuru Kwa Njenga, and those whose parents or guardians did not agree to give consent. Children who had infections other than the intestinal nematodes did not meet the inclusion criteria and were not enrolled in this study.

### 2.5. Collection of Stool Samples

The containers used for stool specimen collection were labeled clearly with the following: subject's name, date of collection, and time the stool was passed. The guardians or parents of study subjects were given a plastic cup with tight fitting lid and two applicator sticks and instructed not to contaminate the samples with urine or toilet water. The fresh stool samples were collected in clean and dry plastic containers. Areas of stool which appeared bloody, slimy, or watery were sampled. If the stool appeared formed, small amounts from each end and the middle were sampled. The lids of the sample containers were tightened to prevent leakage. The samples were then placed in cool boxes and transported to the parasitology laboratory for analysis.

### 2.6. Macroscopic Examination of Stool

As soon as the stool samples arrived in the laboratory, the consistency (degree of moisture) was checked, and one of the following letters was written on the container: F (formed), S (soft), L (loose), or W (watery). If several samples were received at the same time, those containing blood and/or mucus were examined first, followed by liquid specimens. If mucus was present, M was written on the container and recorded on the laboratory note book, and if blood was present, B was written and recorded. Formed specimens were examined last.

### 2.7. Microscopic Examination of Stool Samples

Microscopic analysis of stool samples was done using Kato-Katz technique [10] to assess the intensity of infection. The stool samples were pressed through a mesh screen to remove large particles. A portion of sieved sample was then transferred to the hole of a template on a slide. After filling the hole, the template was removed and the remaining sample covered with a piece of cellophane soaked in glycerol. The glycerol cleared the faecal material from around the eggs. Each slide containing the smear was placed under microscope and the whole area examined in a systematic zigzag pattern. The total number of eggs per slide were recorded for each sample and then converted to eggs per gram (EPG) of stool by multiplying by 24 as was indicated in the information sheet of the Kato set. The value for the individual intensity of infection was obtained according to the Kato-Katz method using arithmetic mean of eggs found on each slide multiplied by 24; each Kato-Katz slide has a capacity of 41.7 mg, so 41.7 mg by 24 is 1000 mg or 1 g [10]. This is the standard measurement to assess the intensity of helminthic infection [11]. This was done in duplicate and mean EPG calculated.

### 2.8. Statistical Analysis

Statistical analysis of numerical data was conducted using one-way ANOVA to determine statistical level of significance in the intensities of nematode infection among the five villages under study as well as comparison of infections between the ages of children (3, 4, and 5 years). The level of statistical significance in all ANOVA analyses was set at *p* < 0.05. The mean egg counts were expressed as mean epg and 95% confidence intervals (95% CIs) were further calculated using Turkey Multiple Comparison test. Intensities of nematode infections were classified into light to heavy intensity of infection according to WHO guidelines [11].

## 3. Results

**Figure 1:**
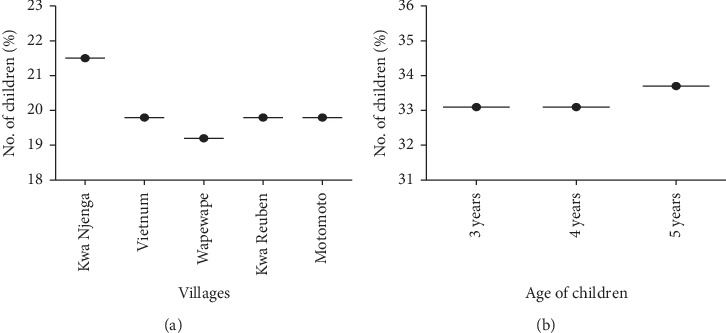
The distribution of children sampled for the study. (a) Distribution of children per village. (b) Age distribution of children.

**Figure 2:**
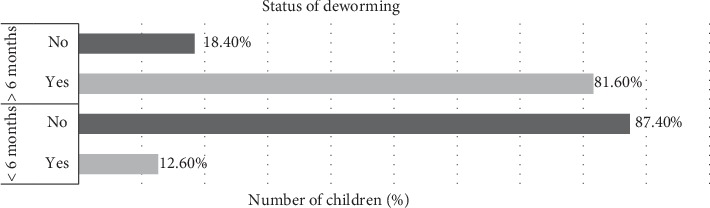
Children dewormed more than 6 months prior to the study. More than 80% of children had been dewormed more than 6 months before the start of this study.

Overall data was collected from 172 children aged 3 to 5 years in 5 villages within Mukuru Kwa Njenga slum settlement in Nairobi County, Kenya. 98 children were sampled during the 1^st^ sampling and 74 children in the 2^nd^ sampling in October 2019. The children had equal representation in terms of gender, age, and distribution within the sampled villages. Of the 172 children sampled, 33.1% were aged 3 years, 33.1% were 4 years, and 33.7% were 5 years (Figure 1). Among the 5 villages, 21.5% of children sampled were in Kwa Njenga, 19.8% in Vietnum, 19.2% in Wapewape, 19.8% in Kwa Reuben, and 19.8% in Motomoto (Figure 1). The study also showed that, in both samplings, averagely 81.6% of children had been dewormed more than 6 months before the study, whereas 12.6% had been dewormed less than 6 months before the study commenced (Figure 2).

### 3.1. Intensity of Intestinal Nematode Infections

**Figure 3:**
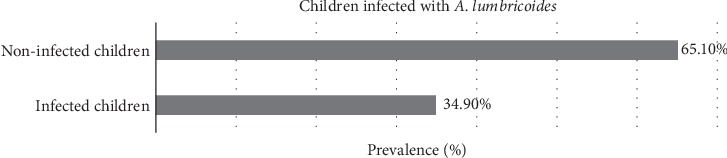
Children infected with intestinal nematodes. Out of 172 children examined for the presence of intestinal nematodes in Mukuru Kwa Njenga settlement, 34.9% were infected with *Ascaris lumbricoides*.

**Figure 4:**
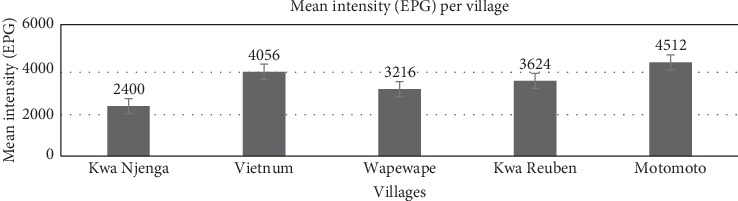
Mean intensity of infection with *A. lumbricoides* in eggs per gram (epg) per village. Sampling was carried out randomly among the five villages of Mukuru Kwa Njenga slum settlement.

**Figure 5:**
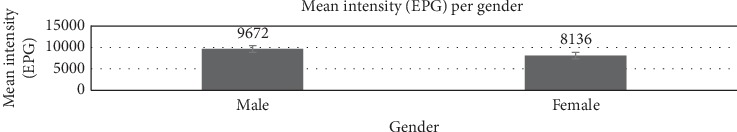
Mean intensity of infection with *A. lumbricoides* in eggs per gram (epg) in the male and female children. Both of the sexes were randomly enrolled in the study and therefore had almost similar representation in distribution within the five villages sampled.

A total of 172 children were examined for the presence of intestinal nematodes, 34.9% were found to be infected with *Ascaris lumbricoides* with a mean infection intensity of 17,808 eggs per gram of stool (epg) (Figures 3 and 4). There was no species of hookworms or *Trichuris trichiura* detected in any of the children sampled. The percentage of female children infected was slightly higher than that of male children; 39.5% of female children were infected with *A. lumbricoides* with a mean infection intensity of 8136 epg, as compared to the 30.2% of male children with a mean infection intensity of 9672 epg (Figure 5). Both of the sexes were randomly enrolled in the study and therefore had almost similar representation.

The distribution of *A. lumbricoides* according to age did not vary markedly. Generally, children aged 3 years had higher number of infections (36.8%) with a mean infection intensity of 6104 epg (95% CI: 5256–6952); 35.1% of the 4-year-olds had mean infection intensity of 5576 epg (95% CI: 4504–6648), whereas the percentage of children infected among the 5-year-olds was 32.8% with a mean infection intensity of 6128 epg (95% CI: 4979–7277) (Table 1).

The prevalence and mean intensities were examined for any intestinal nematodes in five villages within Mukuru slum settlement. Of the 33 participants in Wapewape, 36.3% had *A. lumbricoides* with a mean infection intensity of 3216 epg (95% CI: 2211–4221). Kwa Njenga village had 37 participants; 35.1% were infected with a mean infection intensity of 2400 epg (95% CI: 1107–3693). Vietnum village had 34 participants; 35.2% were infected with a mean infection intensity of 4056 epg (95% CI: 3207–4909). Kwa Reuben and Motomoto villages both had 34 participants each; in both 32.4% had *A. lumbricoides* with varied mean infection intensities of 3624 epg (95% CI: 2899–4349) and 4512 epg (95% CI: 3727–5297), respectively (Table 1).

Statistical comparison of infection intensities using one-way ANOVA and a further analysis using Turkey Multiple Comparison test showed that the level of significance obtained among the five villages was *p*=0.4138, whereas *p*=0.6670 was obtained among the three ages of children. The *p* values were above the set statistical level of significance of 0.05; hence, the intensities of nematode infection were not statistically significantly different in the five villages as well as between ages 3, 4, and 5 years under the study. Age did not affect nematode infection intensities in children. Out of the 34.9% children infected with *A. lumbricoides* in this study, 22.1% had light intensity infections whereas 12.8% had moderate intensity infections (Table 2).

## 4. Discussion

The parasitological analysis of stool samples conducted to detect presence and intensity of infection with *Ascaris lumbricoides*, hookworms, and *Trichuris trichiura* indicated that only one species of intestinal nematodes (*Ascaris lumbricoides*) was found in this study. Thus, the number of species of nematodes infecting children significantly reduced as compared with a previous study in Mukuru where there was prevalence of these three species, and 25% of children under 5 years who presented with diarrhea tested positive for at least one parasite [14]. This indicates that there seem to be a transformation in the epidemiology of the nematode species related to population growth and overcrowded living conditions in urban and slum environments. *A lumbricoides* is one of the STHs that are classified under the neglected tropical diseases (NTDs) that are related to poverty often burdening the communities, but is not given global priority and attention [12]. Individuals with heavy worm burden especially children display associated morbidity with malnutrition, growth stunting, or intestinal obstruction [13].

The overall number of children infected with *Ascaris lumbricoides* did not vary in the 5 villages within Mukuru. A higher prevalence of 36.3% was in Wapewape compared to the other four villages, with a slightly lower prevalence of 32.4% in Motomoto and Kwa Reuben (Table 1). The absence of services such as drainage sanitation and waste management was evident across all the five villages where both samplings were carried out, hence posing health risks for the residents. Most of the households without regular waste collections also indicated diarrhea as one of the reasons for visiting hospitals in the past 6 months. Comparing infection intensities in children diagnosed with *Ascaris lumbricoides* showed no statistically significant difference among the sampled children in this study (*p*=0.4138). The strength of infections depends on the size and nutritional status of the host. It is possible that persistent infection with a particular nematode can impair physical and mental growth [6] and also affect the nutritional status and general development of children [7]. There is evidence that treating worms can lead to improvements in growth and nutritional status.

**Figure 6:**
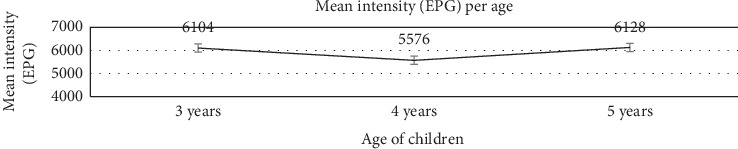
Mean intensity of infection with *A. lumbricoides* in eggs per gram (epg) in the three different ages of children.

Out of the children positive for *Ascaris lumbricoides* in this study, 3-year-olds had a slightly higher infection intensity (Figure 6), indicating that younger children were likely to be more exposed, increasing their chances of contamination than the older children, although the patterns of infection were not significantly different in all the three different age groups (*p*=0.6670). In 2016 Strathmore University Household Finance Survey, over 12% respondents in Mukuru stated they lacked food, thus leading to undernutrition affecting mostly the younger children. Also, faster transmission of these parasites is facilitated by households lacking cemented floors, absence of health and hygiene education, deficiency of uncontaminated channeled water, ill sustained latrines, and children walking without shoes [6]. Although urbanisation can stimulate access to health facilities and public works, congestion and poor hygiene will lead to higher contamination rates through faster closeness of the infested to larger susceptible inhabitants [17].

The higher prevalence of *Ascaris lumbricoides* and higher mean infection intensities in the younger children observed in this study showed that the patterns of infection were not statistically significantly different in age comparisons (*p*=0.6670). The findings of this study show similar results to those reported by Ngonjo et al., 2016, in a study to assess status of soil-transmitted helminthes infections (STHs) in children in Kakamega County in Kenya. The children did not harbor multiple infections with STHs. The mean infection intensity of the nematodes for 22.1% children infected was less than 4999 epg and is categorized as light infection, whereas 12.8% of children infected with *A. lumbricoides* had a moderate infection intensity as was indicated by infection intensity between 5000 epg and 49999 epg (Table 2). The range of mean infection intensities therefore is classified as light to moderate infections according to WHO [14] and Montresor et al. [15].

The absence of other intestinal nematodes in this study indicated a possible success with deworming programs since more than 80% of children had been dewormed more than 6 months before the start of this study (Figure 2). However, there was no elaborate previous related information on deworming programs in this study area that could have been used for comparison with this study to justify low nematode counts in children.

One of the limitations of this study was that there was only one stool sample per individual and this may have influenced the outcome of the prevalence data. The overall outcome of the parasites present may have also been influenced because of the Kato-Katz technique used. While this technique is the most widely used diagnostic method in epidemiologic surveys and drug efficacy trials pertaining to intestinal schistosomiasis and soil-transmitted helminthiasis, its sensitivity is low especially for light infections. Examination of multiple stool samples has been shown to reduce this error [16]. For this study duplicate samples per slide were observed and mean epg calculated.

## 5. Conclusion and Recommendation

The congested living conditions continue to expose children to parasitic infections. *Ascaris lumbricoides* is the predominant intestinal nematode infection in children in Mukuru Kwa Njenga slum settlement. Children are often the reservoir, contributing to continued maintenance of transmission; hence, they remain to be the target of disease control interventions; as a result, public contribution to health education in order to ensure continuous uptake of health services and control of infectious diseases is still significant.

## Figures and Tables

**Table 1:** Prevalence and intensities of infection with *A. lumbricoides*.

	Prevalence (%)	Mean intensity of infection (epg)
Villages		
Kwa Njenga	35.10% (95% CI: 26.40–45.64)	2400 (95% CI: 1107–3693)
Vietnum	35.20% (95% CI: 26.54–45.98)	4056 (95% CI: 3207–4909)
Wapewape	36.30% (95% CI: 27.42–46.34)	3216 (95% CI: 2211–4221)
Kwa Reuben	32.40% (95% CI: 23.36–43.24)	3624 (95% CI: 2899–4349)
Motomoto	32.40% (95% CI: 23.38–43.32)	4512 (95% CI: 3727–5297)

Age of children		
3 years	36.80% (95% CI: 26.44–47.52)	6104 (95% CI: 5256–6952)
4 years	35.80% (95% CI: 25.40–45.42)	5576 (95% CI: 4504–6648)
5 years	33.80% (95% CI: 24.22–44.28)	6128 (95% CI: 4979–7277)

Gender		
Male	30.20 (95% CI: 20.32–41.13)	9672 (95% CI: 8567–10777)
Female	39.50 (95% CI: 30.40–49.64)	8136 (95% CI: 7072–9200)

**Table 2:** Children in each category of *A. lumbricoides* infection intensity.

Category of infection intensity	Children per category (%)
No infection, epg = 0	65.10% (95% CI: 56.09–75.21)
Light intensity, epg > 1 and epg < 4999	22.11% (95% CI: 14.20–32.80)
Moderate intensity, epg > 5000 and epg < 49 999	12.79% (95% CI: 03.80–22.68)
Heavy intensity, epg > 50 000	0.00%

## Data Availability

The field data (obtained from parasitological analysis of stool samples from 172 children aged three to five years living in Mukuru Kwa Njenga slum settlement) used to support this study are available from the corresponding author upon request.
